# Addition to “Er-Doping Enhances the Oxygen Evolution Performance of Cobalt Oxide in Acidic Medium”

**DOI:** 10.1021/acscatal.5c02503

**Published:** 2025-04-22

**Authors:** Sanjiang Pan, Hang Li, Tianyi Wang, Yang Fu, Shenao Wang, Zishuo Xie, Li Wei, Hao Li, Nan Li

In the postpublication review of their 2024 paper, the authors identified three critical methodological improvements: (1) adoption of a more appropriate Shirley background for XPS data fitting; (2) acknowledgment of ongoing academic controversies regarding oxygen vacancy descriptions; (3) implementation of refined analytical methods for quantitative cobalt valence state analysis.

While the conclusions of the original manuscript remain unaffected, certain annotations and statements need to be addressed to ensure a more scientifically rigorous XPS analysis.

**Page 13818, “Results and Discussion”, Sixth Paragraph:** The published text reads “Figure 3c depicts the Co^3+^/Co^2+^ ratios of 0.97 for 4% Er–Co_3_O_4_ and 0.59 for Co_3_O_4_, emphasizing a positive correlation between the Co^3+^/Co^2+^ ratio and catalytic OER activity under acidic media.” Here, the discussion on the quantitative analysis of Co valence states should be revised to focus on qualitative analysis.

**Figure 1:**
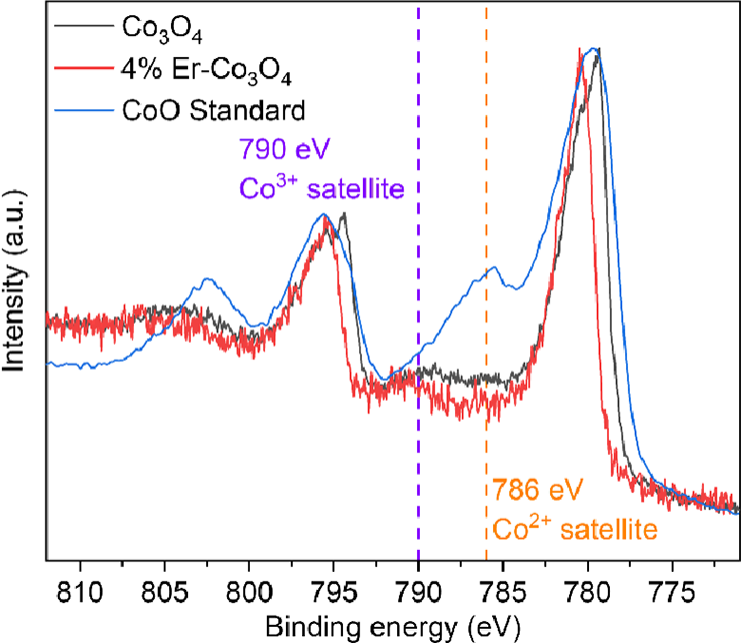
Data graph for High-resolution Co 2p XPS spectra of 4% Er–Co_3_O_4_, Co_3_O_4_ and CoO.

- “As shown in Figure 1, the comparison of high-resolution Co 2p XPS spectra between 4% Er–Co_3_O_4_, Co_3_O_4_, and the CoO standard revealed that the satellite peak of Co^2+^ at 786 eV exhibited significant suppression in Er–Co_3_O_4_. Meanwhile, the satellite peak of Co^3+^ at 790 eV demonstrated a markedly increased proportion in Er–Co_3_O_4_, indicating that Er doping effectively enhances the Co^3+^ content in Co_3_O_4_.”

**Page 13818, “Results and Discussion”, Sixth Paragraph:** The published text reads “As Figure 3b shows, the O 1s spectrum contained two distinct oxygen species, including the Co–O bond (labeled as O1) and the oxygen vacancy site (labeled as O2).^41,44^ The ratios of O2/O1 in 4% Er–Co_3_O_4_ and Co_3_O_4_ are 2.63 and 1.69, respectively (Figure 3d).” “Overall, the increase of Co^3+^/Co^2+^ and O2/O1 in the sample reconfirmed that Er incorporation increased the lattice defects of Co_3_O_4_, exposing more oxygen vacancies and more high-valence Co ions.” The definition of O2 should be revised to “oxygen defects and hydrates”. Additionally, the O2/O1 ratios require adjustment to 2.70 (A) and 1.56 (B) due to the application of Shirley background subtraction in spectral fitting.

**Figure 2:**
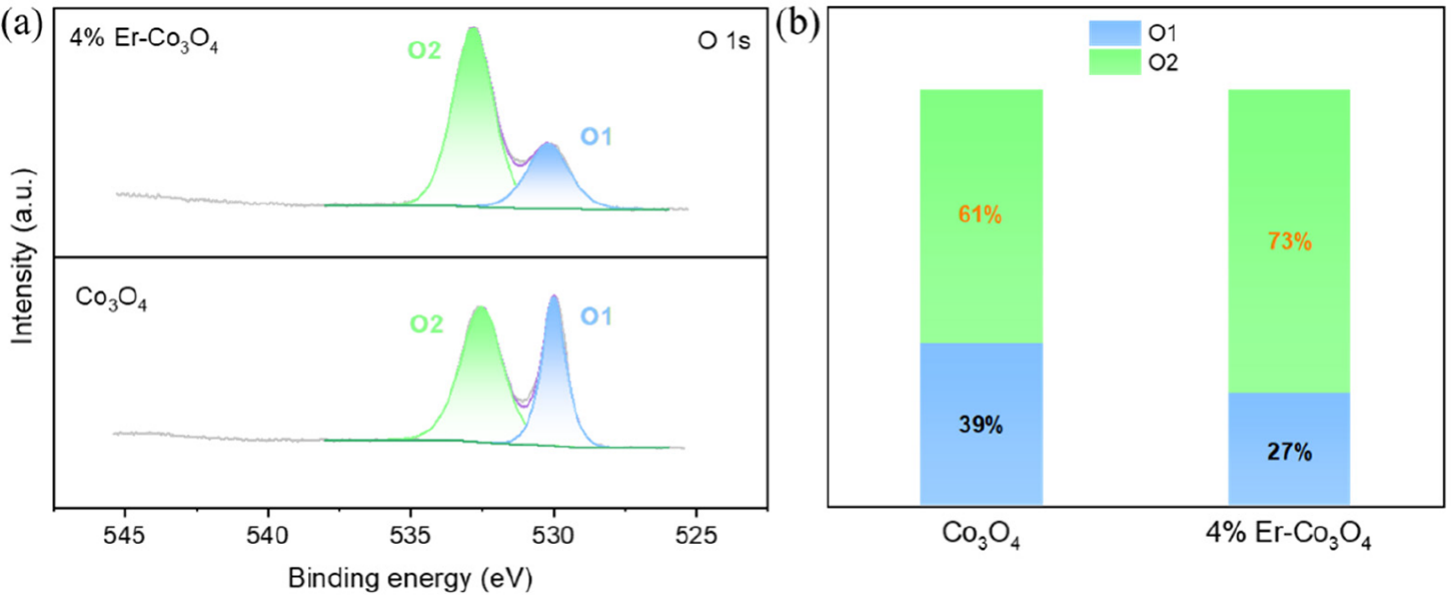
Data graph for High-resolution O 1s XPS spectra of 4% Er–Co_3_O_4_ and Co_3_O_4_ with Shirley-type background.

- “As Figure 2a shows, the O 1s spectrum contained two distinct oxygen species, including the Co–O bond (labeled as O1) and the oxygen defects and hydrates (labeled as O2). The ratios of O2/O1 in 4% Er–Co_3_O_4_ and Co_3_O_4_ are 2.70 and 1.56, respectively (Figure 2b).”
- “These findings are consistent with the results obtained from HR-TEM. Overall, the increase of Co^3+^/Co^2+^ and O2/O1 in the sample reconfirmed that Er incorporation increased the lattice defects of Co_3_O_4_, exposing more oxygen defects and hydrates and more high-valence Co ions.”

